# Origami‐Mediated Low‐Voltage Electret Soft Robotic Actuators for Human‐Machine Haptic Interfaces

**DOI:** 10.1002/advs.75712

**Published:** 2026-05-20

**Authors:** Han Chen, Yongcheng He, Jingyi Liu, Yutong Yuan, Jianhua Li, Yizhuo Qian, Kai Wang, Jian Jiao, Raye Chen‐Hua Yeow

**Affiliations:** ^1^ Department of Electronic and Electrical Engineering Southern University of Science and Technology Shenzhen China; ^2^ Peng Cheng Laboratory Shenzhen China; ^3^ Department of Mechanics and Aerospace Engineering Southern University of Science and Technology Shenzhen China; ^4^ Southern University of Science and Technology Shenzhen China; ^5^ Shenzhen International Graduate School Tsinghua University Shenzhen China; ^6^ Department of Biomedical Engineering National University of Singapore Singapore Singapore

**Keywords:** electret‐based actuation, human‐machine interfaces, low‐voltage haptic feedback, origami‐inspired soft actuators

## Abstract

Soft robotic actuators often require relatively high driving voltages, which limit their portability, safety, and compatibility with compact electronic systems in wearable haptic interfaces. Achieving strong electromechanical coupling at low voltage while maintaining mechanical compliance remains a key challenge for soft actuator design. Here, we present an origami‐mediated low‐voltage electret soft robotic actuator that integrates mechanical compliance and electrical functionality within a symmetric multilayer architecture. Two double‐layer fluorinated ethylene propylene (FEP) electret films with enclosed micro air‐cavity arrays are positioned on both sides of a folded copper origami structure. The origami layer acts as a compliant electrode with a tunable spring‐like response, while the air‐cavity arrays promote high surface potential and stable charge retention. By jointly optimizing electret charging and origami stiffness, the actuator produces perceptible vibrotactile feedback at driving voltages as low as 20 V and supports reliable tactile digital recognition at 70 V using a 7‐segment actuator array. Stable output and durability are maintained over 10 h of high‐frequency operation. Application in a virtual reality piano training task further demonstrates statistically significant improvements in motor learning performance and perceived immersion. This approach offers a compelling pathway toward compact, low‐voltage human‐machine haptic interfaces with robust tactile performance.

## Graphical abstract

This work introduces an origami‐mediated soft electret actuator that combines compliant mechanical design and enhanced charge retention for low‐voltage vibrotactile feedback. The device delivers reliable tactile cues, demonstrates durable performance under continuous actuation, and enables intuitive interaction in applications such as tactile information rendering and immersive virtual reality training, showcasing its potential for compact human‐machine haptic interfaces.

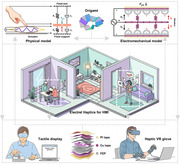

## 1 Introduction

Embodied intelligence and human‐in‐the‐loop robotic systems rely on the coordinated integration of visual, auditory, and tactile modalities to enable natural and intuitive human‐machine interaction [1, 2, 3]. While vision and audition have benefited from rapid advances in sensing, computation, and display technologies, tactile feedback remains comparatively underdeveloped in soft, skin‐interfaced systems due to voltage, stiffness, and scalability constraints [4, 5, 6, 7, 8]. The absence of effective tactile cues not only limits immersion in virtual environments but also constrains assistive applications that depend on spatially resolved tactile communication, such as structured tactile information display and skill learning through touch.

Conventional haptic feedback technologies are predominantly built upon rigid actuation mechanisms, such as eccentric rotating mass motors, linear resonant actuators, and related approaches [9, 10, 11, 12]. Although these systems provide stable vibration output, their bulky form factors and limited mechanical compliance impede seamless integration with soft, conformable, and body‐interfaced platforms. In response, a range of soft electroactive polymers [13, 14, 15], such as dielectric elastomers [16, 17], ionic polymer‐metal composites [18, 19], and piezoelectric polymers [20, 21], have been explored as flexible alternatives. However, practical deployment of these materials in wearable haptics remains hindered by high driving voltages, restricted output force, or long‐term reliability concerns, which raise both safety and system‐level integration challenges. This work addresses a critical gap: scalable, low‐voltage, mechanically compliant haptic actuators with quantified perceptual and task‐level benefits (comprehensive benchmarking against prior works in Table S1).

Electret materials enable low‐voltage electromechanical actuation by sustaining quasi‐permanent charge storage, which allows strong electrostatic interactions to be generated under relatively low applied bias [22, 23, 24]. Building on this characteristic and inspired by traditional origami concepts (Figure S1), we propose an origami‐mediated low‐voltage electret soft robotic actuator that integrates electret‐based electrostatic actuation with a mechanically tunable origami structure [25, 26]. The design combines a compliant origami element with a double‐layer electret architecture incorporating enclosed air cavities, enabling coordinated regulation of mechanical response and charge stability within a compact soft actuator.

In such systems, the achievable actuation performance is fundamentally governed by the charge storage capability and stability of the electret materials. Most existing designs rely on single‐layer electret films or simple multilayer stacking, or employ non‐deterministic void structures, which provide limited control over charge distribution and electric field configuration [27, 28, 29, 30, 31]. In single‐layer configurations, the limited interfacial area restricts charge trapping capacity, while direct exposure to environmental factors accelerates charge decay. By contrast, the double‐layer electret architecture proposed in this work incorporates a deterministic and hermetically sealed micro air‐cavity array, which introduces additional charge‐trapping interfaces and a partially isolated microenvironment, leading to enhanced charge storage capability and improved charge retention stability. This structured design also facilitates stronger electric field generation through field superposition within the cavities and is specifically engineered for stable and efficient actuation under practical mechanical loading conditions, offering a practical structural strategy for electret‐based soft actuators.

**FIGURE 1:**
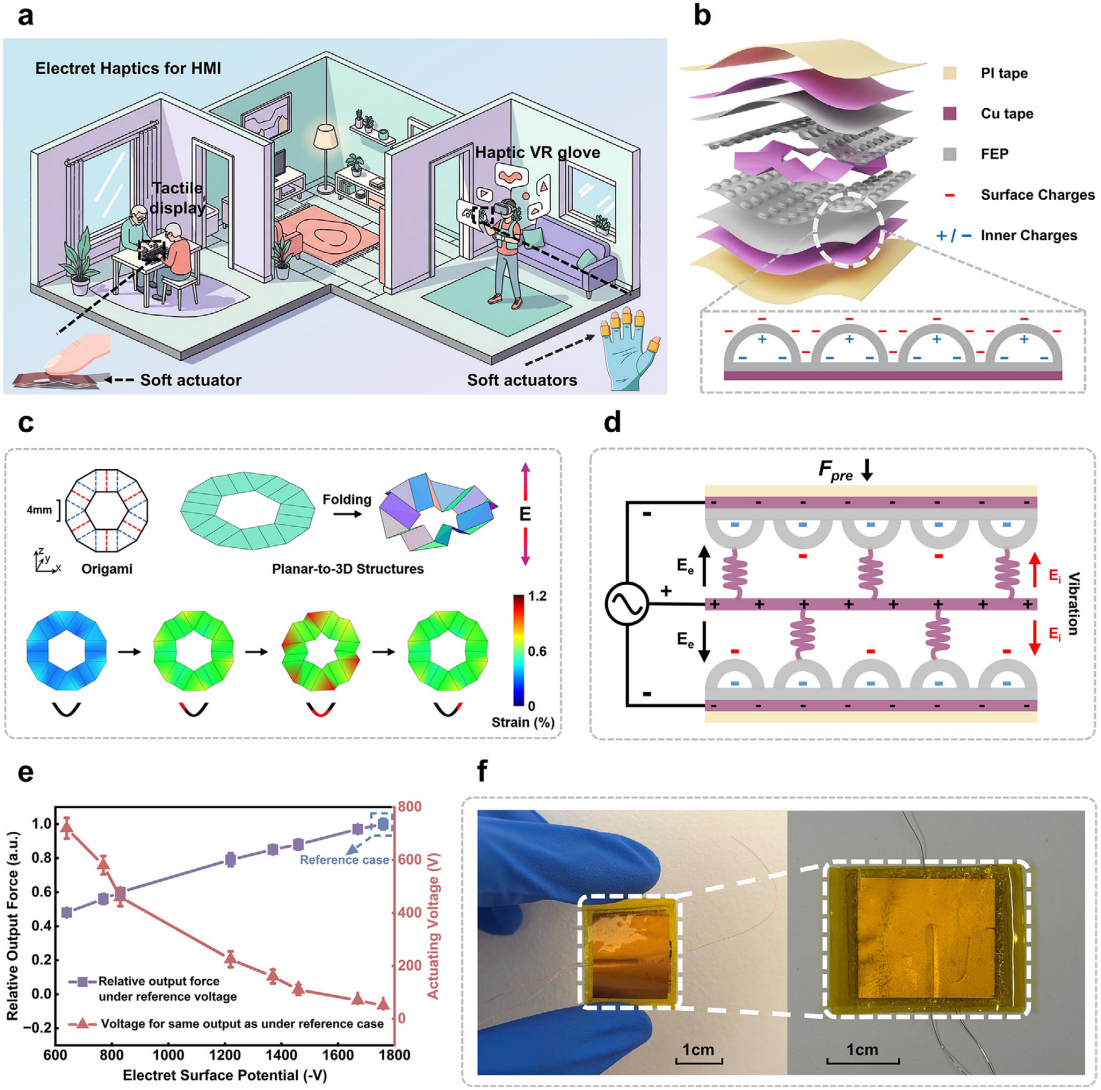
Overview of the origami‐mediated electret soft robotic actuator for low‐voltage human‐machine haptic interfaces. (a) Conceptual design of electret actuator‐based human‐machine interface systems for tactile information display and virtual reality (VR) interaction. Bottom left: schematic illustration of a prototype actuator being pressed by a volunteer's fingertip. Bottom right: schematic of a haptic glove integrating five actuators, each mounted on a fingertip. (b) Schematic illustration of the actuator structure. Two double‐layer FEP electret films incorporating enclosed micro air‐cavity arrays are symmetrically arranged on both sides of a folded copper origami structure. Conductive copper tapes serving as negative electrodes are laminated outside the electret layers, while polyimide (PI) films are applied as the outermost layers for electrical insulation. A dashed box indicates the region enlarged to illustrate the charge distribution on the FEP surfaces and within the enclosed air cavities. (c) Schematic of the folded copper origami middle‐layer structure. Upper left: geometry of the regular dodecagonal origami pattern, showing mountain folds (red lines), valley folds (blue lines), cut lines (black lines), and overall dimensions (outer edge length: 4 mm). Upper right: planar layout and deformation behavior of the origami structure. Bottom: structural simulation of the contraction and corresponding Cauchy strain distribution during the negative actuation phase. (d) Simplified electromechanical model of the actuator. With the fingertip providing a preload, the folded copper origami core is modeled as an equivalent spring. The built‐in electric field *E_i_* generated by the electret charges superimposes with the external electric field *E_e_* induced by the applied alternating voltage. The alternating resultant field produces a time‐varying electrostatic force, driving vibrotactile motion of the soft actuator. (e) Relative output force of the soft actuator measured under a peak‐to‐peak driving voltage of 50 V at different electret surface potentials (purple curve), and the peak‐to‐peak driving voltage required to achieve the same output force as the reference case under varying surface potentials (red curve). (f) Photograph of the fabricated actuator in bent (left) and flat (right) configurations.

From the perspective of tactile perception, the proposed actuator is tailored to efficiently stimulate Meissner and Pacinian corpuscles, which are primarily responsible for dynamic and vibratory sensations in human skin (Figure S2) [32, 33, 34, 35]. Consistent with this design objective, the actuator exhibits the following characteristics: (i) perceptible vibrotactile sensation can be reliably evoked at a driving frequency of 240 Hz at voltages as low as 20 V; (ii) stable and repeatable actuation is maintained during continuous high‐frequency operation for over 10 h; (iii) at 240 Hz and 70 V, a compact 7‐segment actuator array enables reliable tactile digital recognition; and (iv) the actuator enhances user performance in an immersive virtual reality piano training task through synchronized haptic feedback. Figure 1a summarizes these application scenarios by illustrating the integration of the proposed actuator in structured tactile displays and immersive VR interaction, thereby highlighting its potential role in compact, low‐voltage tactile interfaces.

## 2 Results and Discussion

### 2.1 Device Structure and Working Principle

The overall architecture of the origami‐mediated low‐voltage electret robotic actuator is illustrated in Figure 1b. The device adopts a symmetric multilayer configuration consisting of two electret‐based actuation layers and a centrally positioned origami structure that serves as both a compliant mechanical spring and the positive driving electrode. Each actuation layer consists of a double‐layer fluorinated ethylene propylene (FEP) electret incorporating enclosed microscale air‐cavity arrays. A conductive copper tape is laminated onto the outer face of each electret layer to serve as the negative electrode, and polyimide (PI) films are further laminated on the outside for electrical insulation and mechanical protection. The two electret layers are assembled symmetrically on both sides of the origami core, which is fabricated from a 60‐µm‐thick copper tape, forming a compact actuator with overall dimensions of 20 mm × 16 mm. A schematic overview of the fabrication process is provided in Figure S3, with detailed procedures described in the Experimental Section. The enlarged view in Figure 1b highlights the charge distribution within the double‐layer FEP air‐cavity array after corona charging, where electrostatic charges are stored both on the FEP surfaces and within the sealed air cavities, forming a stable electret configuration suitable for electrostatic actuation [26, 36]. The experimental setup used for corona charging is shown in Figure S4.

The mechanical deformation behavior of the central origami structure under electrical excitation is illustrated via structural simulation in Figure 1c. When driven by a sinusoidal alternating voltage, the origami structure undergoes periodic compression and recovery, acting simultaneously as a deformable electrode and a mechanical spring. Owing to its polygonal geometry, the origami structure exhibits moderate stiffness and stable elastic response with a well‐defined strain distribution, which can be conveniently tuned by adjusting the size of the central cut‐out region during fabrication. This tunability allows the actuator to adapt to different preload and deformation conditions encountered during practical use.

Based on the structural configuration and deformation behavior, the operating mechanism of the actuator can be simplified into the electromechanical model shown in Figure 1d. When the device is pressed by a fingertip during operation, a mechanical preload is applied through the outer PI layer, and the folded origami structure can be modeled as a spring with an equivalent stiffness. The charges stored in the corona‐charged electret layers generate an intrinsic electric field *E_i_*, while the externally applied driving voltage introduces an additional electric field *E_e_*. When a positive voltage is applied to the central origami electrode, and negative potentials are applied to the two outer electret layers, the combined electric field induces an electrostatic force that compresses the origami structure. Upon voltage reversal or reduction, the stored elastic energy in the origami structure drives rapid mechanical recovery. The periodic interplay between electrostatic attraction and elastic restoring force gives rise to high‐frequency vibration, which forms the basis of tactile stimulation. A detailed explanation of the simplified electromechanical model and force generation mechanism is provided in Explanation S1.

The effectiveness of the proposed structural design in reducing the required driving voltage is demonstrated in Figure 1e. As the surface potential of the electret layers increases after corona charging, the actuator achieves the same output force at progressively lower driving voltages, confirming that enhanced charge storage is a practical and effective strategy for low‐voltage actuation. The data shown in Figure 1e were obtained from five independently fabricated actuators. This experimental trend is in good agreement with finite element simulation results (Explanation S2), validating the proposed electromechanical model.

Despite the incorporation of multiple functional components, the actuator maintains excellent mechanical flexibility. As shown in the left image of Figure 1f, the fabricated device can be bent and deformed without structural damage. To evaluate its robustness under realistic usage conditions, the actuator was subjected to repeated manual bending, folding, and mild crumpling for over 200 cycles, simulating handling and deformation during wearable operation. After this testing, the right image of Figure 1f shows that the device retained a clean appearance and stable functionality, indicating good mechanical durability and suitability for flexible haptic applications.

### 2.2 Surface Potential and Charge Retention of Electret Layers

**FIGURE 2:**
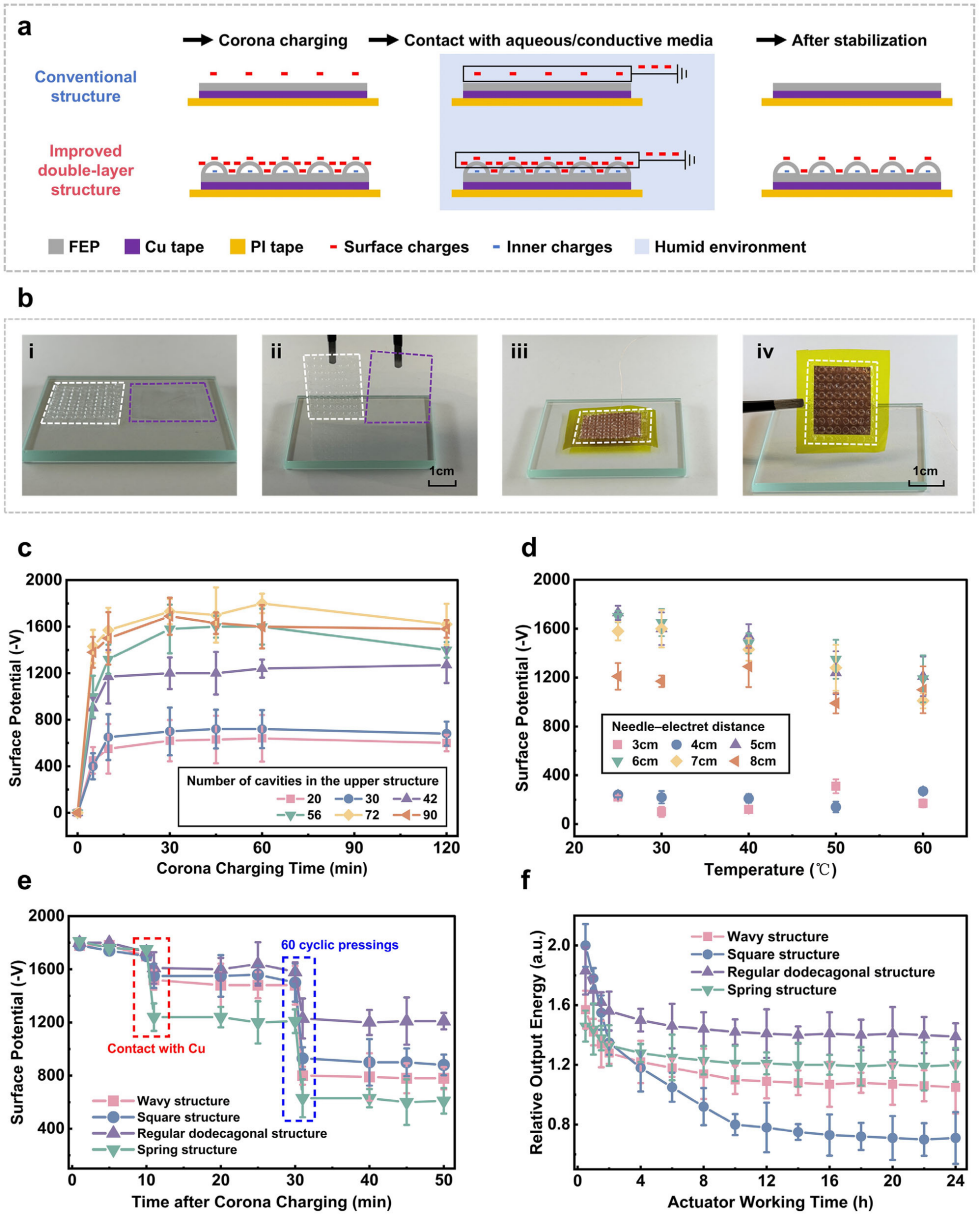
Surface potential and charge retention characteristics of the electret layers. (a) Schematic comparison between a conventional single‐layer FEP electret and the proposed double‐layer FEP electret incorporating enclosed micro air‐cavity arrays, illustrating their different charge distributions and retention mechanisms. (b) Photographs of the fabricated electret structures: (i) top view of a patterned electret and a flat electret placed side by side on a glass substrate; (ii) side view of the same two electrets; (iii) top view of the assembled double‐layer electret structure including the Cu electrode and PI insulation; (iv) side view of the same assembled structure. (c) Evolution of surface potential with corona charging time for electrets with different numbers of air cavities. (d) Surface potential measured under different probe‐sample distances and ambient temperatures. (e) Temporal evolution of surface potential during Cu contact and repeated manual pressing, illustrating charge neutralization and retention behavior after assembly. (f) Relative output energy of actuators employing four different origami geometries versus operating time.

The attainable surface potential largely sets the lower bound of the driving voltage in electret‐based actuators, while long‐term charge stability governs the sustainability of low‐voltage operation. Conventional single‐layer electret films, although thin and flexible, exhibit limited charging efficiency and rapid charge decay under mechanical contact and environmental exposure. To address these limitations, we designed a double‐layer FEP electret incorporating sealed microscale air‐cavity arrays. As illustrated in Figure 2a, under identical corona‐charging conditions, this structure exhibits a markedly higher surface potential and a substantially lower charge decay rate than a single‐layer electret. Quantitatively, the initial surface potential reaches approximately −1.8 kV, compared to −1.4 kV for the single‐layer counterpart, and the charge decay is reduced from 53.9% to 30.8% over 7 days under ambient conditions. During corona charging, the confined air in each cavity undergoes Paschen‐type electrical breakdown, generating free electrons that become trapped on the inner cavity surfaces, forming a population of internal charges in addition to the surface charges on the electret interfaces [37, 38]. Because the cavities are electrically isolated and hermetically sealed, air exchange and charge leakage are strongly suppressed, leading to enhanced charge retention and electrostatic stability. A photograph of the fabricated air‐cavity array is shown in Figure 2b.

Beyond charge storage, the arrayed air‐cavity architecture helps maintain the structural integrity of the electret layer under realistic preload conditions. When finger pressure is applied, the distributed hemispherical cavities share the applied load, which reduces local stress concentration and limits excessive deformation. Compression tests indicate that after a sustained preload of 5 N for 1 h, most air cavities remain intact without noticeable collapse (Figure S5). Moreover, the cavities are oriented toward the interior of the actuator rather than toward the contacting surface, preventing direct fingertip contact with the cavity regions and further reducing the risk of mechanical damage and charge loss during repeated use.

To quantitatively evaluate the factors governing corona charging performance, a series of parametric experiments were conducted. Throughout this work, all electret samples were charged using a negative corona probe operated at an applied voltage of −20 kV. Figure 2c shows the evolution of surface potential with increasing charging time under fixed corona‐charging conditions, with a probe‐to‐sample distance of 5 cm at room temperature and ambient indoor humidity. For each air‐cavity configuration, ten independently prepared samples were tested. A rapid rise in surface potential is observed during the initial charging stage, followed by saturation after approximately 30 min. Extending the charging duration beyond this point does not lead to further improvement and may even result in a slight decrease in surface potential, likely due to enhanced charge recombination or localized discharge events. Among the tested configurations, the structure containing 72 (8 × 9) air cavities achieved the highest surface potential. A possible explanation for this observation, related to the balance between surface area enlargement and electric‐field uniformity, is discussed in Explanation S3.

Building on these observations, we further investigated how probe‐to‐sample distance and temperature influence charging performance while keeping other conditions constant—specifically, a fixed charging duration of 30 min, the same electret type containing 72 air cavities, and ambient indoor humidity without active regulation. These parametric measurements were conducted using five independently prepared samples for each tested condition. As summarized in Figure 2d, decreasing the probe‐to‐sample distance does not lead to a monotonic improvement in charging efficiency. When the probe is positioned too close to the electret surface, excessive local electric fields may induce internal dielectric breakdown or charge leakage, resulting in a low net surface potential. An optimal distance of approximately 5 cm was identified under the present experimental conditions, corresponding to a critical electric field strength below the breakdown threshold. It should be noted that this optimal distance is not universal and depends on multiple parameters, including the applied high‐voltage level and ambient conditions. Temperature‐dependent measurements further reveal that elevated temperatures do not enhance charging efficiency as expected. While mild heating around 30 °C can temporarily increase surface potential, this effect is likely dominated by reduced ambient humidity rather than intrinsic thermal activation [39]. At higher temperatures, increased electron mobility may accelerate charge escape, leading to reduced charge retention. A comprehensive discussion of these influencing factors [40] is provided in Explanation S4.

The origami geometry strongly influences electret charge retention during device assembly and operation, even though it does not affect the corona charging process itself. Four representative origami structures with increasing folding complexity were therefore evaluated (Figure S6). For each origami design, the charge‐retention measurements in Figure 2e were obtained from ten independently prepared samples, while the cyclic mechanical tests in Figure 2f were performed using five assembled actuators. As shown in Figure 2e, a pronounced drop in surface potential occurs immediately after assembly, caused by partial charge neutralization at the contact interface between the origami electrode and the electret layers, and this loss is further amplified under preload and repeated pressing. Among the tested designs, the regular dodecagonal origami (RDO) exhibits the highest charge retention, maintaining a surface potential of approximately −1200 V after cyclic mechanical interaction. This behavior is attributed to its geometry, which restricts electrical contact to discrete points and thereby minimizes the effective charge‐neutralizing area. At the same time, Figure 2f shows that the RDO provides the most stable mechanical response under long‐term cyclic loading, sustaining sufficient restoring force without collapse or stiffness degradation. By comparison, softer structures deform readily but deteriorate rapidly, whereas stiffer designs preserve their shape but yield limited displacement and electrostatic work. The combined advantages of controlled contact geometry and mechanical stability make the RDO the most suitable origami core for preserving electret charge and supporting efficient actuation; the associated output energy is analyzed in the following section.

### 2.3 Mechanical Properties and Preload‐Dependent Actuation Behavior

**FIGURE 3:**
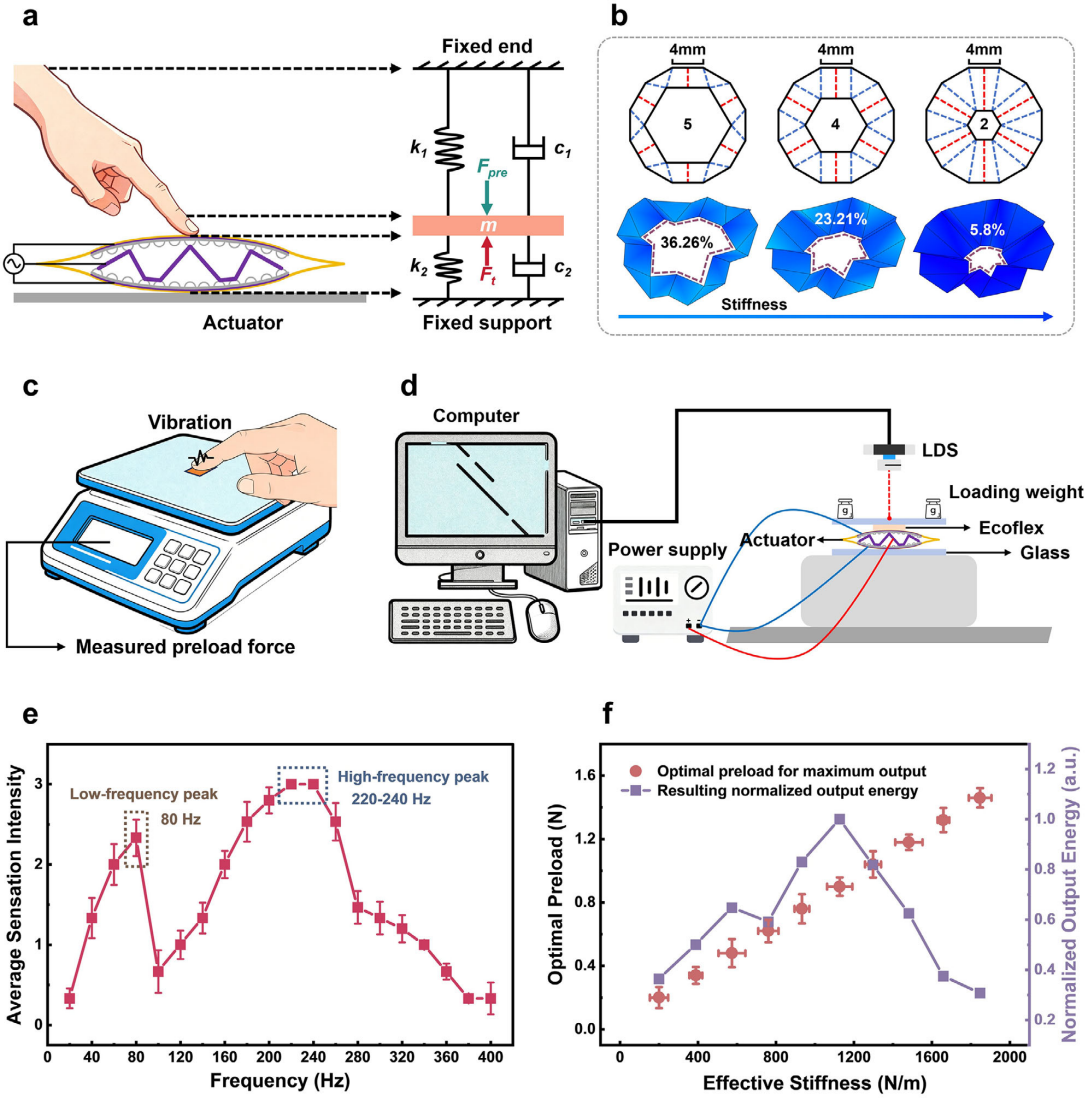
Influence of mechanical stiffness and preload on actuator performance. (a) Equivalent lumped physical model of the finger‐actuator system under electrical excitation. (b) Schematic illustration of origami structures with varying central cut‐out sizes used for stiffness tuning. (c) Schematic illustration of the subjective measurement used to determine the perceptually optimal preload range for an actuator with a given effective stiffness. (d) Experimental setup for measuring actuator vibration displacement under a controlled, fingertip‐like preload. (e) Average sensation intensity at different driving frequencies under a constant driving voltage. (f) Optimal preload force for actuators with different effective stiffnesses and the corresponding normalized mechanical output energy.

When the actuator is pressed by a fingertip, a normal preload is applied, and the resulting finger‐actuator interaction can be simplified into a lumped physical model, as illustrated in Figure 3a, in which the preload and the stiffness of the origami structure jointly determine the vibration amplitude and the resulting tactile output [36, 41, 42, 43, 44]. A detailed description of the modeling assumptions is provided in Explanation S5. Under these conditions, the peak mechanical output energy can be estimated from the applied preload force *F_pre_* and the measured vibration displacement *Y_s_*, as:

(1) $$\Delta W_{peak}=2F_{pre}\cdot Y_{s}$$

The mechanical stiffness of the origami is a key parameter governing the actuator performance, as it directly influences the achievable displacement under preload and therefore the mechanical output energy. The effective stiffness is determined by the geometric configuration of the origami structure, including folding angles, initial height, and cut‐out size. In this work, the folding configuration (i.e., folding angles and overall geometry) was fixed to ensure a consistent structural framework, and the stiffness was therefore tuned by varying the central cut‐out size. To quantify this effect, origami structures with identical folding patterns but different cut‐out sizes were fabricated, as depicted in the schematic in Figure 3b. The corresponding stiffness values are summarized in Figure S7, showing a clear monotonic decrease with increasing cut‐out area. This trend arises from the reduced load‐bearing cross‐section and enhanced fold compliance at larger cut‐out sizes, enabling a versatile range of mechanical responses for different application scenarios. Photographs of representative origami structures are provided in Figure S8 to illustrate the structural variability, and a detailed analysis of geometric effects is given in Explanation S6.

A combined subjective‐objective evaluation was conducted under identical driving voltage and frequency to determine the optimal preload for each origami stiffness and to select the actuator stiffness that produces the strongest tactile sensation, integrating participant perception with quantitative mechanical measurements. 16 participants sequentially evaluated actuators with different origami stiffnesses, adjusting the applied preload for each configuration to identify the range eliciting the strongest tactile perception and scoring the perceived intensity on a 0–3 scale, as schematically illustrated in Figure 3c, which depicts the subjective measurement procedure for a given actuator stiffness. The preloads identified in this subjective evaluation were then applied in a controlled mechanical characterization setup, in which a 4‐mm‐thick Ecoflex 00–30 layer emulated the compliant properties of human skin, calibrated weights were placed on the actuator to reproduce the selected preloads, and a glass substrate ensured uniform load transfer and a rigid reference for displacement tracking. Vibration displacement under each preload was recorded with a laser displacement sensor, and the corresponding mechanical output energy was calculated according to Equation (1), as shown in Figure 3d, which illustrates the experimental setup for measuring actuator vibration displacement. Comparison of the calculated output energies across different stiffness configurations enabled the selection of both the actuator stiffness and the associated preload that provide the strongest tactile feedback, thereby integrating subjective perception with objective energetic output.

Prior to the preload‐stiffness evaluations, the operating frequency was selected based on a separate perception test. 16 participants rated vibrotactile intensity over a frequency range from 20 to 400 Hz at a constant driving voltage of 100 V. As shown in Figure 3e, two sensitivity peaks were observed in the low‐ and high‐frequency ranges, consistent with the known frequency response of human mechanoreceptors [45]. Among the tested frequencies, 240 Hz yielded the strongest and most stable perceptual response and was therefore adopted as the fixed operating frequency for all subsequent measurements. In addition, prolonged vibration was observed to induce a gradual reduction in fingertip sensitivity, requiring higher driving voltages to maintain the same perceived intensity, indicative of sensory adaptation [46, 47], with the detailed results presented in Figure S9.

With the operating frequency fixed at 240 Hz, optimal preloads determined, and corresponding vibration displacements measured, actuators of different stiffness configurations were systematically evaluated. The resulting output energy, derived from these measurements, is summarized in Figure 3f. In this figure, the preload‐force data were obtained from 16 participant evaluations, while the effective stiffness values were averaged from five independently fabricated actuators for each configuration. The maximum mechanical output energy was achieved for an origami with outer and inner edge lengths of 4 mm and a folded height of approximately 2–3 mm. Under this condition, the actuator exhibited a stiffness of 1127 N/m, an optimal preload of 0.9 N, and a vibration amplitude of 2.47 µm. This configuration also produced the strongest subjective tactile sensation reported by participants, with the corresponding data presented in Figure S10. To further validate the combined subjective‐objective procedure, an additional experiment was conducted using actuators with this optimal stiffness. Normalized output energy was measured across a range of driving frequencies and applied preloads. The results, presented in Figure S11, confirm that the maximum output energy occurs at the previously determined optimal preload of 0.9 N and a driving frequency of 240 Hz, thereby objectively corroborating the accuracy of the subjective evaluations.

### 2.4 Actuation Performance and Stability

**FIGURE 4:**
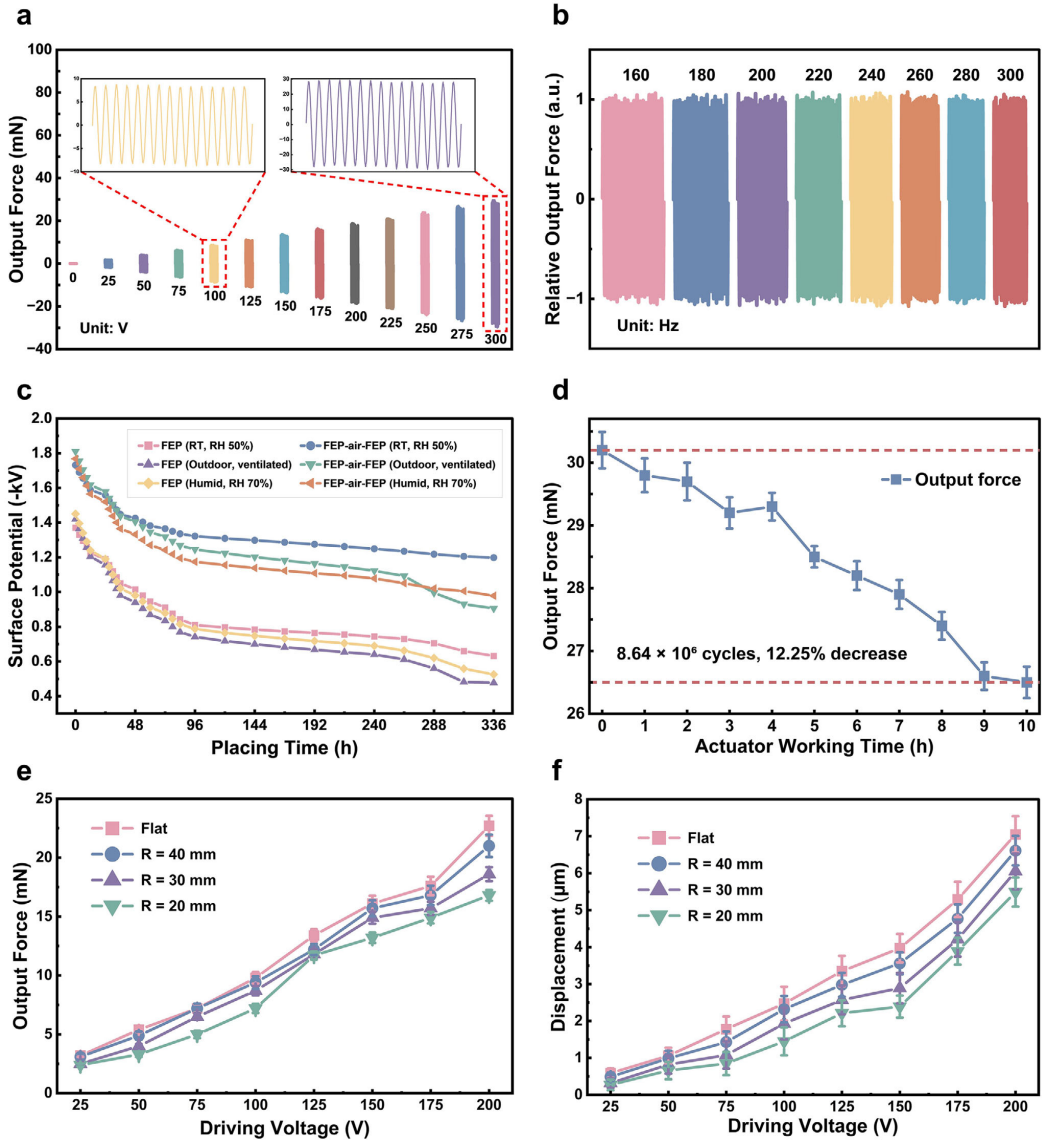
Actuation force and operational stability of the actuator. (a) Output force of the actuator measured at different driving voltages under 240 Hz excitation. (b) Relative output force measured at different driving frequencies under a fixed driving voltage of 100 V. (c) Surface potential decay of single‐layer and double‐layer FEP electrets under different environmental conditions. (d) Long‐term cyclic stability test of the actuator, showing the evolution of output force over 8.64 × 10^6^ actuation cycles. (e) Output force versus driving voltage under different curvature conditions. (f) Vibration displacement versus driving voltage under different curvature conditions.

The output force of the actuator was characterized at 240 Hz under different driving voltages, as shown in Figure 4a. It increases monotonically with applied voltage, indicating stable and controllable electromechanical actuation. At 100 V, the actuator produces an output force of approximately 10 mN, which is sufficient to generate clearly perceivable vibrotactile stimulation at this operating frequency. To examine frequency dependence, the driving voltage was fixed at 100 V, and the excitation frequency was swept from 160 to 300 Hz in 20 Hz steps, with the results shown in Figure 4b. Within this range, the measured output force varies only weakly with frequency under low‐voltage operation. In contrast, human perception tests reveal pronounced frequency‐dependent differences in perceived tactile intensity. This observation indicates that force amplitude alone does not fully determine haptic perception and highlights the importance of matching actuator operation to the frequency‐dependent characteristics of human mechanoreceptors. Meanwhile, the actuator exhibits a fast response time of approximately 3.2 ms and low power consumption at the milliwatt level (0.034–4.42 mW over 20–200 V), further supporting its suitability for low‐voltage and wearable applications (see Explanation S7 for details).

The operational stability of the actuator was evaluated from two perspectives: surface charge retention and cyclic durability. Figure 4c compares the surface potential decay of single‐layer and double‐layer FEP electrets under different environmental conditions. Six test groups were examined, including room temperature at 50% relative humidity, outdoor ventilated conditions, and a humid environment at 70% relative humidity, for both single‐layer and double‐layer structures. For each structure under each environmental condition, six independently prepared samples were tested. The double‐layer FEP electret with air‐cavity arrays consistently exhibits superior charge retention compared with the single‐layer counterpart. A comparative study further confirms that the double‐layer actuator delivers a consistently higher output force than its single‐layer counterpart under identical driving conditions, as detailed in Figure S12. Under ambient indoor conditions, the surface potential decreases relatively rapidly during the first three days, followed by a much slower decay and eventual stabilization. In contrast, high‐humidity and ventilated environments significantly accelerate charge dissipation for both structures. These results indicate that maintaining a dry operating environment is critical for preserving actuator performance and further confirm the effectiveness of the double‐layer air‐cavity design in stabilizing stored charges. The corresponding output stability of the complete actuator under different humidity conditions is presented in Figure S13.

The mechanical durability of the actuator was assessed through long‐term cyclic testing, as shown in Figure 4d. Three independently assembled actuators were evaluated under the same accelerated testing protocol. To accelerate aging effects and obtain a conservative estimate of durability, the actuator was continuously driven at an elevated voltage of 300 V and a frequency of 240 Hz for 10 h. The maximum output force was recorded at 1 h intervals. After approximately 8.64 × 10^6^ actuation cycles, the output force decreased from 30.2 to 26.5 mN, corresponding to 87.75% retention of the initial force. Under these accelerated driving conditions, the actuator demonstrates robust mechanical resilience and long‐term operational stability, while maintaining a substantial force output after nearly ten million cycles.

Additional experiments were conducted on curved substrates with different radii of curvature (20–40 mm) to further evaluate the actuator performance under practical wearable conditions, as illustrated in Figure S14. The output force and vibration displacement under different driving voltages at 240 Hz are summarized in Figure 4e,f, respectively. For each curvature condition, measurements were obtained from five independently assembled actuators. As the curvature increases, both output force and displacement exhibit a gradual reduction. This performance degradation can be attributed to bending‐induced pre‐stress and increased structural constraint, which limit the effective deformation space of the origami structure. Under strong bending conditions (20 mm radius), the actuator retains approximately 70%–80% of its initial performance, indicating robust electromechanical output even under significant deformation. Cyclic tests under different curvature conditions further demonstrate stable operation over prolonged actuation, with only a slight increase in performance degradation at higher curvature (Figure S15). Overall, the actuator maintains reliable vibrotactile output across a wide range of bending conditions, confirming its suitability for conformal and wearable applications.

### 2.5 Digital Recognition Based on Actuator Arrays

**FIGURE 5:**
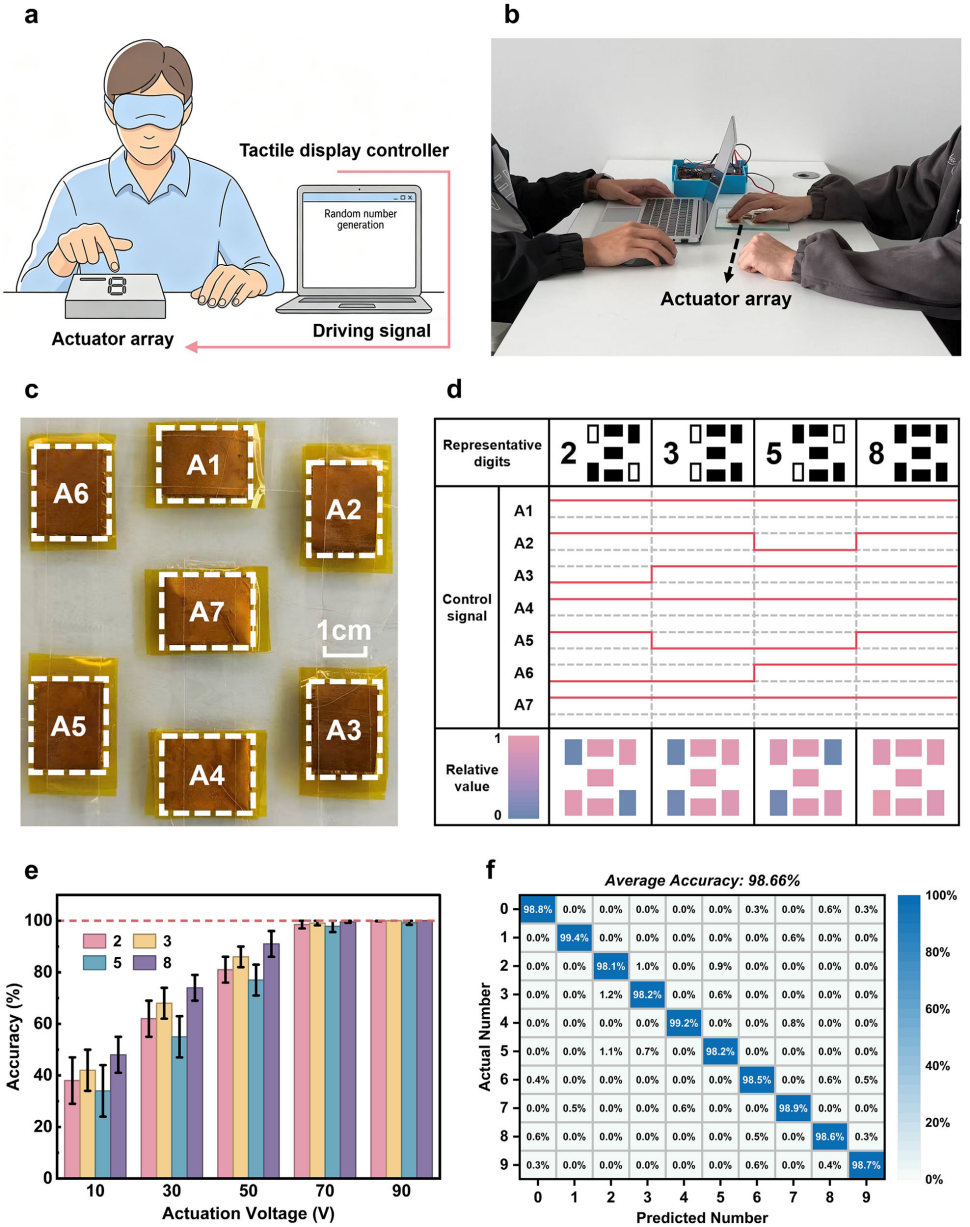
Digital recognition using the origami‐mediated electret actuator array. (a) Schematic illustration of the experimental workflow for tactile digital recognition. (b) Photograph of the tactile recognition experiment, where a participant interacts with the actuator array through fingertip exploration. (c) Photograph of the fabricated 7‐segment actuator array. (d) Driving signals and the corresponding relative output force of each actuator unit for representative digits. (e) Recognition accuracy of four representative digits at different driving voltages. (f) Confusion matrix for ten digits at 70 V, showing an average recognition accuracy of 98.66%.

A tactile digital recognition system based on a 7‐segment actuator array was designed and evaluated to demonstrate the practical feasibility of the proposed origami‐mediated electret actuators in structured information representation. The overall experimental workflow is schematically illustrated in Figure 5a, showing the process from input signal generation to actuator array response.

As shown in Figure 5b, the tactile recognition experiment was conducted with one participant exploring the actuator array using fingertip contact, while the input signals were externally controlled. A magnified view of the fabricated actuator array is presented in Figure 5c. Each actuator unit corresponds to one segment in the 7‐segment display, which is activated or deactivated by controlling the presence or absence of vibration.

Prior to formal human perception experiments, the actuator array was first characterized to verify its ability to reliably reproduce predefined segment patterns. Four representative digits (2, 3, 5, and 8) were selected for validation. These digits include both structurally similar patterns (e.g., 2, 3, and 5) and a fully activated configuration (8), providing a stringent test of spatial discrimination capability. The corresponding control signals and relative output force distributions demonstrate a clear contrast between activated and non‐activated segments, ensuring reliable tactile distinction, as shown in Figure 5d.

With the system validated, perception experiments were conducted with 16 participants to determine the minimum driving voltage required for accurate digital recognition. Four representative digits (2, 3, 5, and 8) were selected to evaluate recognition performance. The results are shown in Figure 5e, where the recognition accuracy increases steadily with driving voltage, reaching nearly 100% at around 70 V. This result confirms that the actuator array can support reliable tactile perception under low‐voltage operation.

After establishing the operating voltage, a more comprehensive recognition experiment was performed using all ten digits (0–9). An independent group of 16 new participants with no prior exposure to the task was recruited for this evaluation. Participants were not informed in advance which digits would appear and were instructed to identify them based on the perceived vibration patterns. All tests were conducted at 70 V and 240 Hz. The results, summarized in Figure 5f, show an average recognition accuracy of 98.66%. In addition to the high recognition accuracy, a clear learning effect was observed, with participants identifying digits more rapidly as the number of trials increased, indicating that the vibrotactile patterns can be efficiently learned with minimal training.

### 2.6 Virtual Reality Demonstration With Haptic Feedback

**FIGURE 6:**
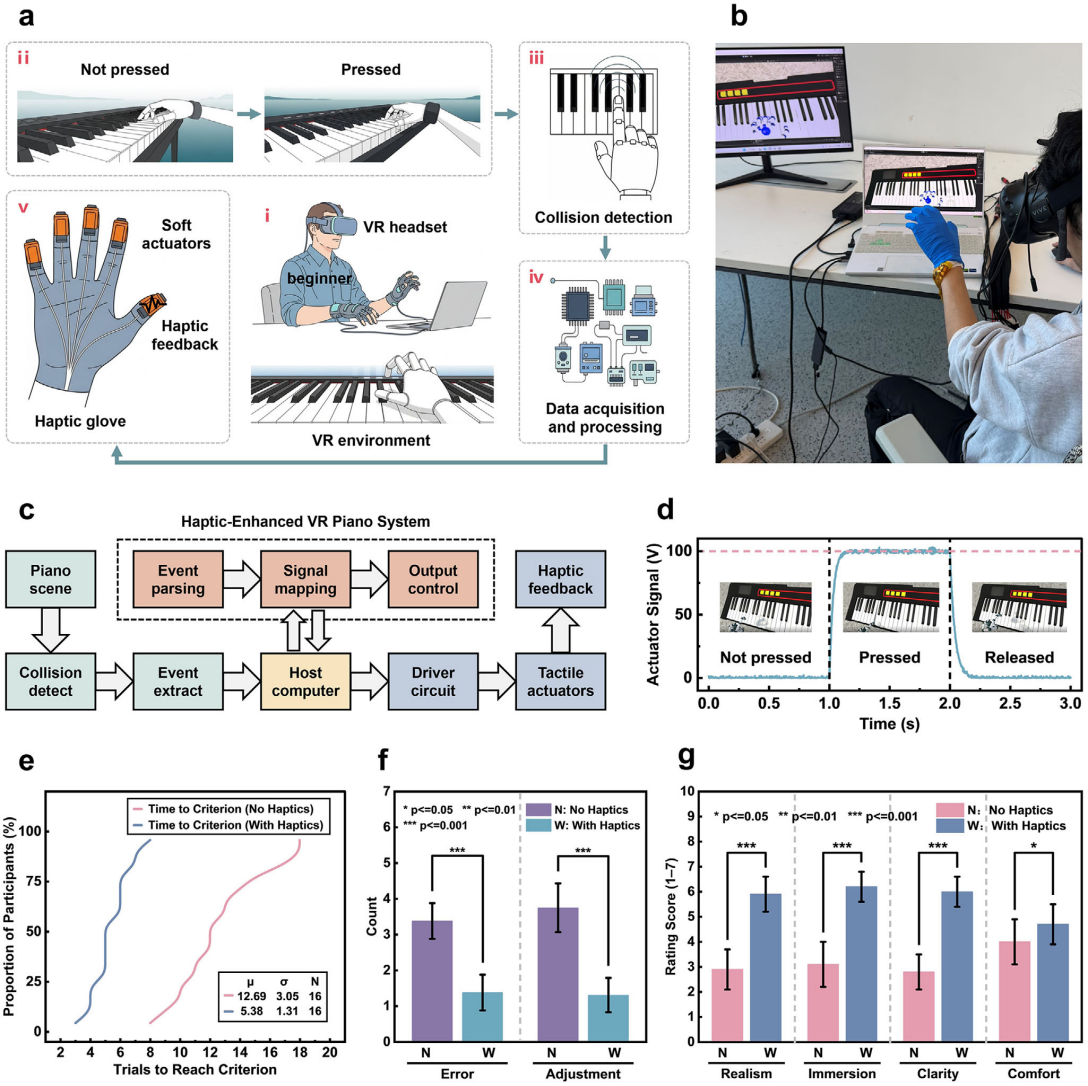
VR‐based piano training experiments. (a) Schematic illustration of the VR piano training principle and experimental workflow. (b) Photograph of a participant performing the VR piano task while wearing the tactile glove. (c) Flowchart of the VR piano training procedure. (d) Driving voltage waveform applied to the fingertip actuators before and after a virtual key press. (e) Cumulative distribution of trials required to reach the learning criterion, plotted as the proportion of participants versus trials to reach criterion, with and without tactile feedback. (f) Experimental results showing the number of note errors and corrective actions, with statistical significance evaluated by paired t‐tests. (g) Subjective participant ratings on realism, immersion, clarity, and comfort under conditions with and without tactile feedback; statistical significance was evaluated by paired t‐tests.

A VR‐based piano training system was established to evaluate the proposed actuator in an immersive interaction task. The overall experimental concept is illustrated in Figure 6a. Participants with no prior piano training wore a VR headset and were immersed in a virtual piano environment. When a participant pressed a virtual piano key, the system detected the collision event and recorded the spatial information of the interaction. This information was processed by a host computer and converted into voltage control signals, which were transmitted to the corresponding fingertip‐mounted actuators. As a result, a brief vibration was delivered to the finger synchronously with the virtual keypress, simulating the tactile sensation of contacting a real piano key. A photograph of a participant performing the VR piano experiment is shown in Figure 6b, and the overall experimental process flow is summarized in Figure 6c. The tactile interaction in the VR piano task is also demonstrated in Video S1. Because the actuators were attached directly to the fingertips and the preload could not be actively controlled during operation, the driving voltage was set to 100 V to ensure robust and perceivable tactile feedback. The corresponding driving signal waveform is shown in Figure 6d.

Two behavioral experiments were used to quantify the impact of tactile feedback on learning performance. In the first experiment, 16 participants performed up to 20 repetitions of a fixed eight‐note sequence in a VR piano environment, with a tempo of 250 ms between successive notes. For each repetition, performance was evaluated based on whether the entire eight‐note sequence was executed correctly. A performance criterion was defined as achieving three consecutive fully correct sequences. The time‐to‐criterion (TTC) was defined as the trial index at which the third consecutive correct sequence was completed; if a participant failed to reach the criterion within 20 trials, the TTC was assigned a value of 21. The resulting TTC distributions, summarized in Figure 6e, show that participants reached the criterion substantially faster when tactile feedback was enabled, with a TTC of 5.38 ± 1.31 trials, compared with 12.69 ± 3.05 trials without haptic feedback, demonstrating a marked acceleration of motor learning induced by fingertip vibrotactile cues.

In the second experiment, the same 16 participants were instructed to perform a simple melody in the VR piano environment, and their performance was evaluated under both haptic and non‐haptic conditions. For each trial, two metrics were extracted: the number of incorrectly played notes and the number of corrective actions required to complete the melody. As summarized in Figure 6f, tactile feedback significantly improved task execution. The mean number of note errors decreased from 3.38 ± 0.50 without haptic feedback to 1.38 ± 0.50 with haptic feedback, and the number of corrective actions was reduced from 3.75 ± 0.68 to 1.31 ± 0.48. Paired statistical comparisons confirmed that both reductions were highly significant (*p* <0.001), indicating that vibrotactile feedback led to more accurate execution with fewer corrective movements.

Following completion of both experiments, these participants provided subjective ratings on a 7‐point Likert scale covering four dimensions: realism, immersion, clarity, and comfort, with the results summarized in Figure 6g. The inclusion of tactile feedback led to substantial increases in perceived realism (from 2.9 ± 0.8 to 5.9 ± 0.7, *p* <0.001), immersion (from 3.1 ± 0.9 to 6.2 ± 0.6, *p* <0.001), and clarity (from 2.8 ± 0.7 to 6.0 ± 0.6, *p* <0.001). Comfort also increased slightly (from 4.0 ± 0.9 to 4.7 ± 0.8, *p* = 0.02), indicating that the addition of tactile feedback did not introduce noticeable discomfort. These results suggest that the proposed haptic feedback enhances the subjective quality of VR interaction across multiple perceptual dimensions without compromising user comfort.

## 3 Conclusions

This work presents an origami‐mediated low‐voltage electret soft robotic actuator capable of stable vibrotactile feedback at low driving voltages by jointly optimizing surface charge retention and mechanical stiffness. A double‐layer FEP electret with sealed microcavity arrays ensures robust surface potential stability, while the origami spring provides tunable stiffness to match typical fingertip preload. At an optimal stiffness of 1127 N/m and a preload of 0.9 N, the actuator achieves maximum mechanical output; it produces a vibration amplitude of 2.47 µm at 240 Hz and delivers a clear tactile sensation at 70–100 V. The output force remains above 87% of its initial value after 8.64 × 10^6^ actuation cycles, demonstrating both strong mechanical durability and surface charge retention.

In application‐level demonstrations, a 7‐segment actuator array achieves 98.66% recognition accuracy for digital patterns at 70 V, and a VR piano‐training system demonstrates enhanced immersion through fingertip haptic feedback. These results highlight the advantages of the proposed actuator in terms of low‐voltage operation, spatially addressable actuation, and compatibility with wearable tactile interfaces.

Collectively, the proposed approach establishes origami‐mediated electret actuators as a scalable, mechanically programmable, and low‐voltage haptic platform, enabling high‐performance tactile feedback for next‐generation wearable and immersive human‐machine interfaces. Building on these demonstrated capabilities, the present study also highlights several practical considerations for real‐world applications, including certain limitations that warrant further improvement, such as limited environmental robustness against humidity, insufficient long‐term charge retention, and challenges in scalable fabrication for large‐area or high‐density integration.

## 4 Experimental Section

### 4.1 Fabrication of the Actuator

The origami‐mediated low‐voltage electret soft robotic actuator was fabricated through a multilayer lamination and assembly process, as schematically illustrated in Figure S3. Briefly, a conductive copper tape (20 µm thick) was first laminated onto a flat FEP film (20 µm thick) to serve as the outer electrode layer. In parallel, a second FEP film was patterned into a microwell array using a pressing process with complementary 3D‐printed molds. The patterned FEP film was then aligned and bonded to the flat FEP/Cu film, forming an enclosed micro air‐cavity array sandwiched between the two FEP layers. Subsequently, a PI tape (20 µm thick) was laminated onto the outer surface of the copper electrode to provide electrical insulation. The resulting FEP/air‐cavity/FEP/Cu/PI laminate was subjected to corona charging, during which electrostatic charges were generated and accumulated on the FEP surfaces, including those associated with the enclosed air cavities, yielding a stable electret structure. For the mechanically compliant core, a thin copper tape (60 µm thick) was patterned into a regular dodecagon with a central hexagonal cutout and folded along predefined crease lines to form the three‐dimensional origami structure. Finally, two charged electret laminates were assembled symmetrically on both sides of the folded copper origami element, resulting in a complete actuator with overall dimensions of 20 mm × 16 mm.

### 4.2 Experimental Setup and Characterization

The corona charging system consisted of a direct current power supply (LRS‐75‐12), a high‐voltage module (KDHM‐Q1‐12S20000N‐VI), a grounded metal plate, and an acrylic protective enclosure. The surface potential of the electret samples was characterized using an electrostatic voltmeter (JSGONG GT‐4). Actuator vibration displacement was measured with a laser displacement sensor (ST‐P25EA), while the output force was quantified using a force measurement system comprising a load cell (DSX‐306) and a universal force measuring instrument (D054). A compact wireless driving system, employed to enable portable operation and independent multi‐channel actuation, is schematically illustrated in Explanation S8.

For the virtual reality experiments, a commercial VR setup was used in combination with optical hand tracking (Leap Motion Controller 2) to enable real‐time interaction with a virtual piano environment developed in Unity 3D. Finger motion was continuously tracked and processed by the physics engine to detect collision events between the virtual fingertips and piano keys. Upon collision, the spatial information of the interaction was mapped by the host computer to the corresponding fingertip‐mounted electret actuators. Control commands were transmitted wirelessly via Bluetooth to the custom driving circuit, which generated high‐voltage excitation signals to drive the actuators. In principle, the interaction parameters such as impact force, contact velocity, and contact area could be mapped to voltage amplitude, driving frequency, and actuator selection, respectively. To simplify the experimental design and ensure consistency across participants, all VR experiments were conducted using a fixed driving condition of 100 V and 240 Hz once a collision event and actuator location were determined.

All measurements in this study were independently repeated multiple times using separately prepared samples or devices to ensure reproducibility. The number of replicates varied depending on the specific experiment and is described in the corresponding Results sections. Experimental data are presented as mean values, and error bars represent standard deviation unless otherwise specified.

## Funding

This work was supported by the Ministry of Education Academic Research Fund Tier 1 (A‐8004048‐00‐00), the Major Key Projects of PCL under Grants (PCL2025A17‐2, PCL2025A12‐2), and the Guangdong S&T Program under Grant (2024B0101010003).

## Conflicts of Interest

The authors declare no conflicts of interest.

## Supporting information




**Supporting File 1**: advs75712‐sup‐0001‐SuppMat.docx.

**Supporting File 2**: advs75712‐sup‐0002‐VideoS1.mp4.

## Data Availability

The data that support the findings of this study are available from the corresponding author upon reasonable request.
