# Supplementary material for: Origami‐Mediated Low‐Voltage Electret Soft Robotic Actuators for Human‐Machine Haptic Interfaces
Source: Adv Sci (Weinh). 2026 May 20;13(45):e75712. doi: 10.1002/advs.75712 (PMC13335916; doi:10.1002/advs.75712)
Supplement: Supplementary file 1 [file ADVS-13-e75712-s002.docx]

Supporting Information

Origami-Mediated Low-Voltage Electret Soft Robotic Actuators for Human-Machine Haptic Interfaces

Han Chen, Yongcheng He, Jingyi Liu, Yutong Yuan, Jianhua Li, Yizhuo Qian, Kai Wang*, Jian Jiao*, and Raye Chen-Hua Yeow*

**Supplementary Figures**

**Figure S1.** Representative traditional origami geometries that inspire the design of enclosed cavity architectures in this work. **(a)** Huffman Extruded Boxes. **(b)** Huffman Waterbombs. **(c)** Huffman Rect Weave. **(d)** Huffman Exdented Boxes. Each origami pattern can be folded to generate a regular array of discrete cavities, which can be converted into sealed air chambers when covered by a flat sheet, providing a conceptual basis for the enclosed air-cavity array adopted in the electret structure.

**Figure S2.** Schematic illustration of the four primary types of cutaneous mechanoreceptors in human skin. **(ⅰ)** Meissner’s corpuscles (FA-I): rapidly adapting receptors sensitive to low-frequency vibrations and light dynamic touch over small skin areas. **(ⅱ)** Merkel’s discs (SA-I): slowly adapting receptors that encode sustained pressure and fine spatial features such as edges and texture. **(ⅲ)** Ruffini endings (SA-II): slowly adapting receptors responsive to skin stretch and sustained deformation. **(ⅳ)** Pacinian corpuscles (FA-II): rapidly adapting receptors highly sensitive to high-frequency vibrations transmitted through deeper tissue layers. The operating frequency range and compliant deformation mode of the proposed actuator primarily target Meissner and Pacinian corpuscles, which are responsible for dynamic and vibrotactile perception in human skin.

**Figure S3.** Fabrication process of the origami-mediated electret soft robotic actuator. **(a)** Step-by-step fabrication procedure. **(ⅰ)** A conductive copper tape (20 µm thick) is laminated onto a flat FEP film (20 µm thick) to form an outer electrode layer. In parallel, a second FEP film is embossed into a microwell array using a pressing process with two complementary 3D-printed molds. **(ii)** The patterned FEP film is aligned and bonded to the flat FEP/Cu film, forming an enclosed micro air-cavity array between the two FEP layers. **(iii)** A PI tape (20 µm thick) is laminated onto the outer surface of the copper electrode to provide electrical insulation. **(iv)** The laminated FEP/air-cavity/FEP/Cu/PI stack is subjected to corona charging, during which electrostatic charges are generated and accumulated on the FEP surfaces, including those facing the enclosed air cavities, leading to the formation of a stable electret structure. **(v)** A thin copper tape (60 µm thick) is patterned into a regular dodecagon (side length: 4 mm), with a regular hexagonal cutout (side length: 4 mm) removed from the center. **(vi)** The patterned copper tape is folded along predefined crease lines to form the three-dimensional origami structure. **(vii)** Two charged FEP/air-cavity/FEP/Cu/PI units are assembled symmetrically on both sides of the folded copper origami structure, yielding a complete actuator with overall dimensions of 20 mm × 16 mm. **(b)** Fabrication steps of the patterned FEP film via the embossing (pressing) process using 3D-printed molds.

**Figure S4.** Experimental setup for corona charging. **(a)** Optical photograph of the corona charging apparatus, consisting of a power supply, a high-voltage module, a corona probe, and a grounded metal plate. **(b)** Schematic illustration of the corona charging configuration used to deposit charges onto the electret layers.

**Figure S5.** Mechanical stability of the double-layer FEP air-cavity electret under preload. **(a)** Optical photograph of the double-layer FEP structure sandwiched between two glass substrates and subjected to a constant compressive load of 5 N for 1 h. **(b)** Optical photograph taken after removal of the load, showing that most air cavities remain intact without noticeable collapse.

**Figure S6.** Representative origami spring geometries evaluated in this study. **(a)** Wavy structure. **(b)** Square structure. **(c)** Regular dodecagonal structure. **(d)** Spring structure.

**Figure S7.** Measured effective stiffness of the origami structure for various hexagon side lengths (n = 5).

**Figure S8.** Optical images of the fabricated origami structures. **(a)** Oblique-view photographs of origami structures fabricated with different materials and dimensions. From left to right: copper foil (4 mm side length), A4 paper (4 mm), A4 paper (6 mm), and A4 paper (10 mm), showing the visual appearance of the structures. **(b)** A copper-foil origami structure (4 mm side length) under compression by a glass plate. The observable deformation confirms its flexibility and deformability.

**Figure S9.** Perceptual adaptation results obtained from 16 volunteers under prolonged vibrotactile stimulation. After continuous adaptation at 80 Hz for 10 min, the sensitivity to low-frequency vibrations was markedly reduced, whereas the sensitivity to high-frequency vibrations showed only a slight decrease. In contrast, after 10 min of adaptation at 240 Hz, a more uniform reduction in sensitivity was observed across the tested frequency band. These results indicate that low- and high-frequency stimulation induce distinct adaptation profiles in human vibrotactile perception.

**Figure S10.** Average sensation intensity reported by 16 participants for actuators with different origami stiffnesses under their respective optimal preload forces, tested at a fixed driving voltage of 100 V and a frequency of 240 Hz.

**Figure S11.** Normalized output energy versus driving frequency under different preload forces for an actuator with an effective stiffness of 1127 N/m, measured at a fixed driving voltage of 100 V (n = 5, mean values shown).

**Figure S12.** Performance comparison of actuators fabricated with different FEP layer configurations. **(a)** Output force (left axis) and displacement (right axis) of the actuators versus driving voltage at 240 Hz (n = 5). **(b)** Time-dependent stability of output force and displacement over a 14-day period for both configurations (n = 5).

**Figure S13.** Normalized output force of the actuator under different relative humidity (RH) environments.

**Figure S14.** Photograph of the actuator mounted on a curved PDMS substrate.

**Figure S15.** Cyclic stability under different curvature conditions during 5 h continuous operation (n = 5).

**Table S1.** Comparison with state-of-the-art haptic actuators.

| Actuator Type | Device Size (mm) | Driving Voltage (V) | Output Force (mN) | Frequency Range (Hz) | Response Time (ms) | Power Consumption (mW) | Operational Stability | Application Scenarios | Ref. |  |
| --- | --- | --- | --- | --- | --- | --- | --- | --- | --- | --- |
| Electromagnetic bistable transducer | Height: 2.1–4.1 mm | / | 1400 (max) | 50–200 | / | Energy: 58 mJ/transition | >1.2×10^5^ cycles | Sensory substitution; balance assist | [1] |  |
| Dielectric Elastomer Actuator (DEA) | 130 × 130 × 13 | 3500 | 600 | 0–300 | 2 (charging); 0.4 (discharging) | / | ~1×10^6^ cycles, negligible degradation | Master-slave tactile communication; in-car navigation | [2] |  |
| Electro-hydraulic soft actuator | / | 12000 | 100 | 1–100 | 150 | / | Stable over 50 cycles (150 s periodic input) | Space robotics; AR | [3] |  |
| DE-based EAP actuator (multilayer) | / | 1900–4000 (forearm); 2300–4000 (fingertip) | 255 (max) | 0–250 (perceptible); up to 500 | <1 | / | <1% force deviation over 10^6^ cycles | Tactile glove; forearm feedback | [4] |  |
| Microstructured pyramidal DEA | Not specified (area: 100 mm^2^; height: 300 μm) | / | / | 100–200 | <2 | / | <15% degradation after 2.5×10^5^ cycles | Wearable haptics | [5] |  |
| DEMA (AgNWs electrode) | 110 × 60 (array); unit: 5.8 × 7.0 | 2000–8000 (5–20 MV/m) | 130 (max) | 20–500 (optimal ~240) | <1 | / | <8% degradation over 10^6^ cycles | Wearable visuo-haptic interface | [6] |  |
| Flexible DE vibrotactile actuator | 36 × 36 × 0.65 | 2000 (AC, V_pp_) | / | 1–300 | ~18 | / | Good reproducibility (variation < JND, 16%) | Foldable displays | [7] |  |
| PVC gel-based EAP actuator | 36 × 36 × 1 | 200–1500 (AC) | / | 1–300 | / | / | Stable cyclic actuation | Wearables; VR haptics | [8] |  |
| HAxEL actuator | 6 × 6 × 0.8 (single); array: 5×5 | ≤1400 | >300 (normal); ≤15 (shear) | ≤200 | <5 | / | ~10^4^ cycles; failure by delamination | VR gloves; soft robotics | [9] |  |
| Piezoelectret actuator/sensor | 10 × 10 (pixel); 20 × 20 (array) | ~500 (for 150 μm, inferred) | >20 | 25–500 | / | / | <1% variation (sensor mode, 6000 cycles) | AR/VR haptics; HMI | [10] |  |
| Origami-mediated electret actuator (This work) | 20 × 16 | 20–100 | 10 (at 100 V) | 1–300 | 3 | 0.034–4.42 | ~10⁷ cycles, stable output | Wearable HMI; AR/VR haptics | This work |  |

**Supporting explanation 1:** Electromechanical model of the electret-based soft robotic actuator

(1) Physical configuration and modeling assumptions

The electret-based soft robotic actuator is modeled as a one-dimensional electromechanical system consisting of two compliant electrodes separated by an air gap and an electret layer connected in series, as shown in **Figure S16**. The upper electrode is supported by an elastic structure that can be approximated as a linear spring with stiffness $k$. Fringing fields, edge effects, and nonlinear mechanical behavior are neglected for analytical clarity. Let the instantaneous electrode separation be $d$, including an air gap of thickness $d_{a}=d-d_{e}$ and an electret layer of thickness $d_{e}$ with relative permittivity $\varepsilon_{e}$. A voltage $V$ is applied across the electrodes, and the electret layer carries a uniform surface charge density $\sigma_{e}$.

(2) Electric field distribution in the layered structure

Due to the one-dimensional configuration and the absence of free volume charges in the dielectric layers, the normal component of the electric displacement field is uniform across the air gap and electret layer. The electric fields in the air gap ($E_{a}$) and electret layer ($E_{e}$) therefore satisfy:

$$\begin{aligned} \varepsilon_{0}E_{a}=\varepsilon_{0}\varepsilon_{e}E_{e}\#\left( 1 \right) \end{aligned}$$

The presence of fixed charges within the electret gives rise to a built-in electric field that exists even in the absence of an externally applied voltage. Under an applied voltage $V$, the total electric field in each layer can be regarded as the superposition of the field induced by the external voltage and the internal field associated with the electret charges.

(3) Voltage-field relationship

The applied voltage is equal to the sum of the voltage drops across the air gap and the electret layer:

$$\begin{aligned} V=E_{a}d_{a}+E_{e}d_{e}\#\left( 2 \right) \end{aligned}$$

Substituting the field relationship yields:

$$\begin{aligned} V=E_{a}\left( d-d_{e}+\frac{d_{e}}{\varepsilon_{e}} \right)\#\left( 3 \right) \end{aligned}$$

Solving for the electric field in the air gap gives:

$$\begin{aligned} E_{a}=\frac{V}{\left( d-d_{e} \right)+\frac{d_{e}}{\varepsilon_{e}}}\#\left( 4 \right) \end{aligned}$$

This expression shows that the layered structure can be characterized by an effective electrical thickness determined jointly by the air gap and electret layer.

(4) Electrostatic force in the absence of electret charges

The electrostatic force acting on the movable electrode is governed by the Maxwell stress in the air gap. The corresponding electrostatic pressure is:

$$\begin{aligned} p=\frac{1}{2}\varepsilon_{0}E_{a}^{2}\#\left( 5 \right) \end{aligned}$$

and the total force is therefore:

$$\begin{aligned} F_{0}=\frac{1}{2}\varepsilon_{0}A\left( \frac{V}{\left( d-d_{e} \right)+\frac{d_{e}}{\varepsilon_{e}}} \right)^{2}\#\left( 6 \right) \end{aligned}$$

This expression reduces to the classical parallel-plate electrostatic force when the electret layer is absent.

(5) Contribution of electret charges

The electret layer introduces a built-in electric field $E_{e}$ in the air gap, originating from the fixed charges stored within the electret. The total electric field in the air gap can therefore be expressed as:

$$\begin{aligned} E_{a}^{\mathrm{tot}}=\frac{V}{\left( d-d_{e} \right)+\frac{d_{e}}{\varepsilon_{e}}}+E_{e}\#\left( 7 \right) \end{aligned}$$

where $E_{e}$ represents the effective electret-induced field. The resulting electrostatic force becomes:

$$\begin{aligned} F=\frac{1}{2}\varepsilon_{0}A\left( \frac{V}{\left( d-d_{e} \right)+\frac{d_{e}}{\varepsilon_{e}}} + E_{e} \right)^{2}\#\left( 8 \right) \end{aligned}$$

Expanding the above expression yields:

$$\begin{aligned} F=\frac{1}{2}\varepsilon_{0}A\left( \frac{V^{2}}{\left[ \left( d-d_{e} \right)+\frac{d_{e}}{\varepsilon_{e}} \right]^{2}} + \frac{2VE_{e}}{\left( d-d_{e} \right)+\frac{d_{e}}{\varepsilon_{e}}} + E_{e}^{2} \right)\#\left( 9 \right) \end{aligned}$$

(6) Physical implications for low-voltage actuation

The resulting force expression contains three distinct contributions: a quadratic voltage-dependent term associated with conventional electrostatic actuation, a linear voltage-field coupling term arising from the electret-induced internal field, and a voltage-independent term determined solely by the electret charges. These additional terms account for the enhanced force output observed under low-voltage excitation. Furthermore, the appearance of an effective electrical thickness explicitly reflects the combined influence of the air gap and electret layer on electromechanical coupling.

**Figure S16.** **(a)** Electromechanical model of the actual actuator. **(b)** Simplified three-electrode parallel-plate model derived from (a). **(c)** Further-simplified electromechanical model used for analysis, obtained from (b).

**Supporting explanation 2:** Numerical analysis of electret-assisted force generation

A simplified finite-element model, as shown in **Figure S17a**, was established in COMSOL Multiphysics 6.3 to analyze electret-assisted electrostatic force generation in the actuator. The model adopts a layered electrostatic configuration consisting of a top electrode, an electret layer, an air gap, and a bottom electrode, with material properties consistent with the device structure. A uniform electric field normal to the electrode surfaces was assumed, while edge effects, fringing fields, and mechanical deformation were neglected to focus on the dominant electrostatic behavior.

The electrostatic field distribution was solved using the Electrostatics interface. The bottom electrode was set as ground, and a fixed external driving voltage was applied to the top electrode. The electret effect was represented by assigning a fixed electric potential to the electret-air interface, corresponding to different electret surface potentials. By parametrically varying this surface potential while keeping all other parameters unchanged, the influence of the electret on electrostatic force generation could be systematically evaluated.

The electrostatic force acting on the top electrode was obtained by integrating the Maxwell stress tensor over the electrode surface and was treated as the equivalent output force of the actuator. The simulation results, shown in **Figure S17b**, indicate that under a fixed applied voltage, increasing the electret surface potential leads to a monotonic increase in the equivalent electrostatic output force. This behavior reflects the enhancement of the electric field strength in the air gap induced by the electret and illustrates the role of electret charging in enabling effective force generation at low driving voltages. Although the model is intentionally simplified and does not account for mechanical deformation or dynamic effects, it captures the essential trend associated with electret-assisted electrostatic actuation.

**Figure S17.** **(a)** Finite element model implemented in COMSOL Multiphysics 6.3. **(b)** Relative output force versus electret surface potential under a fixed applied voltage, normalized to −1000 V.

**Supporting explanation 3:** Effect of air-cavity array density on corona charging performance

The introduction of sealed air-cavity arrays in double-layer electrets significantly influences corona charging behavior. By increasing the internal interfacial area, these cavities facilitate charge accommodation and stabilization, resulting in a rapid rise in surface potential and higher saturation levels for structures with moderate cavity density. However, charging performance does not improve indefinitely with cavity count. In overly dense arrays, reduced cavity dimensions and spacing can perturb the local electric-field distribution. Localized field concentrations near cavity edges and thin ligaments may promote premature charge injection saturation or recombination, limiting effective charge accumulation despite the increased surface area.

Consequently, the 8 × 9 configuration appears to be an optimized regime where the advantages of interfacial area are utilized without triggering significant localized discharge. This mechanism explains why further increasing the cavity number fails to yield higher surface potentials under identical charging conditions. **Figure S18** shows the mold geometries used to define the air-cavity array.

**Figure S18.** **(a)** Mold component with protruding features. **(b)** Mold component with recessed features, where the diameter of the recessed circular openings is designed to be 0.2 mm larger than that of the corresponding protruding features to ensure proper assembly.

**Supporting explanation 4:** Factors influencing corona charging efficiency and stability in electret materials

Corona charging is a complex and multi-parameter process in which the final surface potential of an electret is determined by the combined influence of electrical, geometrical, environmental, material, and process-related factors. No single parameter alone dictates charging performance; instead, reproducible and efficient charge deposition requires an appropriate balance among these contributing elements.

(1) From electrical perspective, the applied high-voltage magnitude, polarity, and stability of the corona source directly affect ion generation and charge injection efficiency. Excessively weak electric fields lead to insufficient ionization, whereas overly strong fields can induce partial discharge, charge recombination, or dielectric damage, thereby limiting net charge accumulation.

(2) Geometrical factors primarily govern the spatial distribution of the electric field and ion flux. Parameters such as probe-sample distance, probe geometry, sample size, and surface topography influence local field uniformity and charging homogeneity. Non-uniform or highly concentrated fields can promote localized discharge and reduce effective surface potential, even under relatively low applied voltages.

(3) Environmental conditions, particularly ambient humidity and temperature, exert a strong influence on corona charging and charge retention. Moisture adsorption on dielectric surfaces facilitates charge leakage through surface conduction pathways, making humidity one of the most critical external variables. Temperature affects charging both directly, by modifying charge mobility and trapping dynamics, and indirectly, through its coupling to humidity and air conductivity.

(4) The intrinsic properties of the electret material further determine its charge storage capability. Dielectric constant, trap density, surface chemistry, and bulk resistivity all influence how injected charges are captured and retained. In multilayer or structured electrets, internal interfaces, voids, or enclosed cavities can introduce additional charge trapping mechanisms, while also altering local electric-field distributions.

(5) Finally, process-related parameters, including charging duration, mechanical handling, and post-charging exposure, affect the attainable and stable surface potential. Charging typically exhibits a saturation behavior, beyond which prolonged exposure yields diminishing returns or even charge loss. Mechanical contact or deformation can induce partial charge neutralization, particularly in soft and flexible electret systems.

Overall, corona charging should be regarded as a coupled electro-environmental-material process rather than a single-step operation. A comprehensive consideration of these factors is therefore essential for achieving reliable, reproducible, and application-relevant electret charging performance.

**Supporting explanation 5:** Preload-regulated response of the electret-based soft robotic actuator

(1) Output energy under fingertip preload

When a fingertip presses against the soft actuator, the interaction involves a preload force $F_{\mathrm{pre}}$ applied by the fingertip and a dynamic force generated by the actuator. For low-voltage soft haptic actuators, the achievable actuation force is inherently limited, whereas stable fingertip contact requires a substantially larger preload; consequently, under practical experimental conditions, the preload force is naturally much larger than the actuator-generated force $\left( F_{\mathrm{pre}} \gg F_{\mathrm{act}} \right)$. In this regime, continuous contact between the fingertip and the actuator is maintained throughout the actuation cycle, and the actuator-induced displacement occurs without appreciably altering the preload force. As a result, the output energy during actuation is primarily determined by the work done by the preload force acting over the actuator-induced displacement, while the contribution from the dynamic actuation force can be neglected without affecting the overall energy scaling.

During one actuation cycle, the fingertip undergoes a displacement with a peak amplitude $Y_{s}$ and subsequently returns under the same preload condition. Accounting for both displacement processes, the peak output energy delivered during the cycle can be expressed as:

$$\begin{aligned} \Delta W_{\mathrm{peak}}=2F_{\mathrm{pre}}\cdot Y_{s}\#\left( 10 \right) \end{aligned}$$

(2) Electromechanical derivation of the optimal preload condition

Under fingertip loading, the electret-based soft robotic actuator is analyzed using the electromechanical model introduced in Supporting explanation 1, with the preload force incorporated as an additional mechanical boundary condition. The initial distance between the electret structure and the bottom electrode, corresponding to the effective thickness of the supporting spring structure in the unloaded state, is denoted as $D_{0}$.

(2.1) Static equilibrium under preload

When a preload force $F_{\mathrm{pre}}$ is applied, the supporting structure undergoes elastic compression until a static mechanical equilibrium is reached. At this stage, the electrostatic force is negligible compared with the preload force, and the force balance can be expressed as:

$$\begin{aligned} F_{\mathrm{pre}}=k\left( D_{0}-D_{1} \right)\#\left( 11 \right) \end{aligned}$$

where $D_{1}$ denotes the compressed distance between the electret structure and the bottom electrode under the applied preload. Therefore, the preload-defined equilibrium gap is given by:

$$\begin{aligned} D_{1}=D_{0}-\frac{F_{\mathrm{pre}}}{k}\#\left( 12 \right) \end{aligned}$$

This equilibrium distance serves as the initial operating point for subsequent electrostatic actuation.

(2.2) Electrostatic force under coupled electrical and mechanical conditions

According to the electrostatic model established in Supporting explanation 1, the actuator can be regarded as a series dielectric system composed of an air gap and an electret layer. The effective electrical thickness is therefore written as:

$$\begin{aligned} D_{\mathrm{eff}}=D_{1}+\frac{t_{e}}{\varepsilon_{e}}\#\left( 13 \right) \end{aligned}$$

where $t_{e}$ and $\varepsilon_{e}$ are the thickness and relative permittivity of the electret layer, respectively. Under an applied driving voltage $V_{\mathrm{app}}$ and an electret-induced equivalent voltage $V_{e}$, the average electric field between the electrodes is:

$$\begin{aligned} E=\frac{V_{\mathrm{app}}+V_{e}}{D_{\mathrm{eff}}}\#\left( 14 \right) \end{aligned}$$

Based on the Maxwell stress formulation, the electrostatic attraction pressure acting between the electrodes is:

$$\begin{aligned} p=\frac{1}{2}\varepsilon_{0}E^{2}\#\left( 15 \right) \end{aligned}$$

and the total electrostatic force can be expressed as:

$$\begin{aligned} F_{\mathrm{elec}}\left( D_{1} \right)=\frac{1}{2}\varepsilon_{0}A\left( \frac{V_{\mathrm{app}}+V_{e}}{D_{1}+\frac{t_{e}}{\varepsilon_{e}}} \right)^{2}\#\left( 16 \right) \end{aligned}$$

This expression highlights the strong nonlinear dependence of the electrostatic force on the electrode separation.

(2.3) Small-displacement approximation and vibration amplitude

When an AC driving voltage is applied, the electrostatic force introduces a periodic perturbation around the static equilibrium position $D_{1}$, leading to oscillatory motion of the compliant electrode. For the operating conditions of the soft actuator, the vibration amplitude is small compared with the equilibrium gap, allowing the use of a small-displacement approximation. Under this approximation, the maximum displacement amplitude can be estimated as the ratio between the electrostatic force and the equivalent stiffness:

$$\begin{aligned} Y_{s,\max}\approx\frac{F_{\mathrm{elec}}\left( D_{1} \right)}{k}\#\left( 17 \right) \end{aligned}$$

Substituting Equation (12) and Equation (16) into Equation (17) yields:

$$\begin{aligned} Y_{s,\max}\approx\frac{1}{2}\frac{\varepsilon_{0}A}{k}\left( \frac{V_{\mathrm{app}}+V_{e}}{D_{0}-\frac{F_{\mathrm{pre}}}{k}+\frac{t_{e}}{\varepsilon_{e}}} \right)^{2}\#\left( 18 \right) \end{aligned}$$

which explicitly relates the actuator displacement amplitude to the preload force.

(2.4) Physical origin of the optimal preload

Equation (18) shows that the preload force influences the actuator response by adjusting the static equilibrium gap. When the preload is small, the equilibrium separation remains large, resulting in a weak electric field and limited electrostatic actuation. As the preload increases, the reduced gap significantly enhances the electric field strength, leading to a rapid increase in electrostatic force and vibration amplitude. However, when the preload becomes excessive, the available gap for dynamic motion is strongly constrained, suppressing periodic displacement despite the presence of strong electrostatic attraction. **Figure S19** illustrates the corresponding model states under four representative preload conditions.

As a consequence, the actuator displacement, output force, and output energy exhibit a non-monotonic dependence on the preload force, giving rise to an optimal preload condition. This behavior originates from the strong gap sensitivity of electrostatic actuation combined with the elastic constraint imposed by the supporting structure.

**Figure S19.** Schematic representations of the actuator model under different preload conditions: **(a)** no preload, **(b)** insufficient preload, **(c)** optimal preload, and **(d)** excessive preload. The preload adjusts the equilibrium distance between the electret structure and the bottom electrode, leading to weak actuation, optimal periodic motion, or constrained motion, respectively.

(3) Model reduction from tactile interaction to an equivalent single-degree-of-freedom system

As illustrated in **Figure S20a**, tactile stimulation involves direct mechanical interaction between the fingertip and the soft electret-based actuator under an applied preload. During operation, the fingertip remains in continuous contact with the actuator surface, and the perceived tactile sensation arises from the vertical vibration of the actuator transmitted to the fingertip.

From a mechanical perspective, this interaction can be initially represented by a coupled system consisting of the fingertip and the actuator structure, as shown in **Figure S20b**. In this representation, the fingertip and the actuator are modeled as two interacting masses, $m_{1}$ and $m_{2}$, connected through elastic and dissipative elements. The fingertip experiences an external contact force $F_{1}$, while the actuator is subjected to the electrostatic driving force $F_{2}$. This configuration forms a two-degree-of-freedom mass-spring-damper system that captures the coupled dynamics of the tactile interface.

Under typical operating conditions, however, a sufficiently large preload force is applied to maintain stable contact between the fingertip and the actuator. As a result, relative motion between the fingertip and the actuator surface is strongly suppressed, and the two bodies move in a mechanically coupled manner. In this case, the two-degree-of-freedom system can be effectively reduced to an equivalent single-degree-of-freedom model, as shown in **Figure S20c**, where the combined mass $m$ represents the coupled fingertip-actuator system.

Within this reduced-order framework, the dominant deformation occurs along the out-of-plane direction of the compliant supporting structure. The system response can therefore be described using a standard mass-spring-damper formulation:

$$\begin{aligned} m\ddot{x}+c\dot{x}+kx=F_{t}\left( t \right)\#\left( 19 \right) \end{aligned}$$

where $x$ denotes the effective vertical displacement, $k$ and $c$ represent the equivalent stiffness and damping of the supporting structure, respectively, and $F_{t}(t)$ is the electrostatic actuation force. This simplified model captures the essential mechanical behavior relevant to tactile output while providing a tractable basis for analyzing actuator displacement, force transmission, and energy output.

**Figure S20.** **(a)** Schematic illustration of the tactile interaction. **(b)** Corresponding physical representation as a two-degree-of-freedom coupled mass-spring system. **(c)** Simplified single-degree-of-freedom physical model used for analysis, where the dominant deformation occurs along the out-of-plane direction of the compliant supporting structure.

**Supporting explanation 6:** Geometric analysis of origami structures

(1) Geometric variability and performance trade-offs

As shown in **Figure S21**, the folding angle ($\theta$) emerges as the pivotal determinant of the system’s initial height ($H$) and the resultant inter-layer gap ($d$). Increasing $\theta$ transitions the structure toward a more planar configuration, thereby reducing the initial gap. While a smaller $d$ theoretically enhances the electrostatic driving force ($F\propto1/d^{2}$), it simultaneously introduces a critical trade-off between interfacial charge stability and mechanical responsiveness.

Experimental data indicate that the attenuation of surface potential post-assembly arises primarily from localized charge neutralization at the contact interfaces between the origami electrodes and the electret layers. This neutralization is exacerbated under sustained preload and repeated mechanical interactions. Specifically, a relatively flatter and lower configuration (large $\theta$) increases the effective contact area, accelerating long-term charge degradation. In contrast, a taller, more three-dimensional configuration (small $\theta$) limits electrical contact to discrete points, effectively preserving surface potential by minimizing the neutralizing surface area.

However, maximizing charge stability via an extremely “pointed” configuration (very small $\theta$) is constrained by the resulting increase in structural stiffness. Such configurations exhibit high mechanical impedance, making the actuator difficult to drive and reducing the vibrational displacement necessary for high-fidelity tactile feedback.

Therefore, the selection of the folding angle is treated as a multi-objective optimization, balancing electrostatic driving force, actuation displacement, and cyclic electrical stability. Based on this analysis, a folding angle of 120° was identified and adopted as the standardized configuration, which harmonizes these competing demands. This geometry-enabled programmability ensures reliable haptic stimulation while maintaining the electromechanical integrity of the device throughout its operational lifespan.

**Figure S21.** Geometric variability. **(a)** Geometry of the regular dodecagonal origami pattern, showing mountain folds (red lines), valley folds (blue lines), and cut lines (black lines). **(b)** Planar layout of the origami structure. $L_{1}$: side length of the regular dodecagon; $L_{2}$: side length of the central hexagon. **(c)** Three-dimensional view of the origami structure, where $\theta$ denotes the folding angle and $H$ represents the structural height. **(d)** Front view of the origami structure, illustrating $\theta$ (folding angle) and $H$ (structural height). **(e)** Simplified schematic of the actuator, where $d$ denotes the maximum vertical separation between the top and bottom FEP layers of the origami structure.

(2) Theoretical basis for geometric modulation of performance

The mechanical response of the origami-mediated interface is fundamentally dictated by an energy-stiffness coupling grounded in the Principle of Virtual Work. By defining the total potential energy ($U$*)* stored within the origami structure—comprising both crease rotation and facet bending—as a function of the generalized actuation coordinate ($z$), we derive the structural stiffness ($K_{eff}$) as the second variation of the system’s energy:

$$\begin{aligned} K_{eff}\left( \theta\right)=\frac{\partial^{2}U\left( \theta\right)}{\partial z^{2}}\approx\sum_{i=1}^{n} k_{i}\left( \frac{\partial\alpha_{i}}{\partial z} \right)^{2}\#\left( 20 \right) \end{aligned}$$

In this governing expression, $k_{i}$ denotes the intrinsic rotational stiffness of the $i$-th crease, while the term $\frac{\partial\alpha_{i}}{\partial z}$ characterizes the kinematic sensitivity—the rate of local angular deformation relative to the global vertical displacement ($z$). This analytical formulation clarifies the physical origin of the stiffness surge observed in small $\theta$ (pointed) configuration topologies: as the geometry becomes more “pointed”, the kinematic sensitivity term intensifies, necessitating a disproportionately higher energy input for incremental displacement. Such a mathematical mapping transitions our design strategy from empirical observation to a predictive framework, enabling the precise “programming” of haptic feedback via strategic geometric modulation.

**Supporting explanation 7:** Response time and power consumption of the actuator

(1) Response time

A quantitative evaluation of the actuator’s dynamic response was carried out by measuring its transient output force under electrical excitation. A gated square-wave signal (240 Hz, 50% duty cycle, 100 V_pp_) was applied to the actuator, and the resulting force was recorded using the measurement system described in the manuscript, consisting of a load cell (DSX-306) and a universal measuring instrument (D054). To accurately capture the high-frequency transient response, the instrument was operated at a sampling frequency of 4800 Hz.

The response time in this study is defined as the interval required for the peak-to-peak vibration amplitude to rise from 10% to 90% of its steady-state value. Based on five independent measurements, the average response time was determined to be 3.23 ± 0.15 ms (the representative time-domain response is illustrated in **Figure S22**).

This rapid onset is equivalent to less than one full vibration cycle at the operating frequency, where $T$ ≈ 4.17 ms. Such performance demonstrates the low mechanical inertia and superior electromechanical coupling inherent in the origami-mediated structure. Furthermore, this high-speed response ensures that tactile cues can be delivered with negligible latency, which is critical for maintaining temporal synchrony in real-time human-machine interaction and immersive virtual reality environments.

**Figure S22.** Raw, unfiltered output force during dynamic response to a gated square-wave signal (240 Hz, 50% duty cycle, 100 V_pp_).

(2) Power consumption

Regarding the power consumption, direct current measurement is challenging due to the low operating current and the influence of parasitic capacitance. Therefore, an analytical approach based on the experimentally measured capacitance and the dielectric loss tangent ($\tan\delta$) was adopted.

The capacitance ($C$) of the actuator was characterized using an LCR meter (VICTOR 4092A). The average power consumption ($P$) was estimated according to:

$$\begin{aligned} P=V_{\text{rms}}^{2}\cdot\omega\cdot C\cdot\tan\delta\#\left( 21 \right) \end{aligned}$$

where $V_{\text{rms}}^{2}$ is the root-mean-square driving voltage and $\omega$ is the angular frequency. For fluoropolymer-based electret materials, $\tan\delta$ is typically reported in the range of 10^-4^ to 10^-3^ in the literature. Considering practical device-level losses, a conservative representative value of 10^-3^ was adopted in this work.^[11–13]^

As shown in **Figure S23**, the power consumption increases quadratically with the driving voltage, ranging from 0.034 ± 0.003 mW at 20 V to 4.42 ± 0.23 mW at 200 V. These results indicate that the actuator maintains low power consumption at the milliwatt level even under relatively high driving voltages.

**Figure S23.** Power consumption versus driving voltage, showing a quadratic dependence.

**Supporting explanation 8:** Compact wireless driving system for multi-channel electret actuation

A compact wireless driving system was developed to support portable operation and independent multi-channel actuation; its system-level architecture is illustrated in **Figure S24**. The main control module is built around an STM32F103ZET6 microcontroller, which is responsible for signal processing, timing generation, and channel control. Wireless communication between the host computer and the driving circuit is achieved using an HC-04 Bluetooth module, supporting real-time data transmission at a rate of up to 60 KB/s. The microcontroller communicates with multiple multiplexer chips via an SPI interface to regulate the switching of low-voltage control signals for individual channels.

The power management module is supplied by rechargeable lithium batteries providing nominal voltages of 3.7 V and 12 V. A multi-stage DC-DC conversion scheme is adopted, consisting of a 3.7 V-to-5 V boost converter followed by a 5 V-to-3.3 V buck converter, generating stable voltage rails of 3.3 V, 3.7 V, 5 V, and 12 V to meet the requirements of the microcontroller, logic circuitry, and high-voltage driving stage.

The high-voltage switching module comprises eight independently addressable output channels. Each channel integrates a high-voltage MOSFET (IXTL2N450) and a dedicated gate-driving circuit, enabling reliable switching at voltages up to 5000 V and operating frequencies up to 600 Hz. This architecture allows independent, high-frequency control of multiple electret actuators in a compact and wearable form factor.

**Figure S24.** System architecture of the compact wireless driving circuit used for independent multi-channel actuation. The system consists of a main control module based on an STM32 microcontroller, a power management module with multi-stage DC-DC conversion, and an eight-channel high-voltage switching module.

**References**

[1]	M. T. Flavin, K.-H. Ha, Z. Guo, S. Li, J.-T. Kim, T. Saxena, D. Simatos, F. Al-Najjar, Y. Mao, S. Bandapalli, C. Fan, D. Bai, Z. Zhang, Y. Zhang, E. Flavin, K. E. Madsen, Y. Huang, L. Emu, J. Zhao, J.-Y. Yoo, M. Park, J. Shin, A. G. Huang, H.-S. Shin, J. E. Colgate, Y. Huang, Z. Xie, H. Jiang, J. A. Rogers, *Nature* **2024**, *635*, 345.

[2]	H. Phung, P. T. Hoang, H. Jung, T. D. Nguyen, C. T. Nguyen, H. R. Choi, *IEEE/ASME Transactions on Mechatronics* **2021**, *26*, 2495.

[3]	J. Ma, X. Cheng, P. Wang, Z. Jiao, Y. Yu, M. Yu, B. Luo, W. Yang, *Applied Sciences* **2020**, *10*, 8827.

[4]	S. Mun, S. Yun, S. Nam, S. K. Park, S. Park, B. J. Park, J. M. Lim, K.-U. Kyung, *IEEE Transactions on Haptics* **2018**, *11*, 15.

[5]	D. Pyo, S. Ryu, K.-U. Kyung, S. Yun, D.-S. Kwon, *Appl. Phys. Lett.* **2018**, *112*, 061902.

[6]	S. Yun, S. Park, B. Park, S. Ryu, S. M. Jeong, K.-U. Kyung, *IEEE Transactions on Industrial Electronics* **2020**, *67*, 717.

[7]	Y. H. Heo, D.-S. Choi, D. E. Kim, S.-Y. Kim, *Applied Sciences* **2021**, *11*, 12020.

[8]	W.-H. Park, Y. Yoo, G. Choi, S. Choi, S.-Y. Kim, in *Haptics: Science, Technology, and Applications* (Eds.: D. Prattichizzo, H. Shinoda, H. Z. Tan, E. Ruffaldi, A. Frisoli), Springer International Publishing, Cham **2018**, pp. 148–156.

[9]	E. Leroy, R. Hinchet, H. Shea, *Advanced Materials* **2020**, *32*, 2002564.

[10]	J. Zhong, Y. Ma, Y. Song, Q. Zhong, Y. Chu, I. Karakurt, D. B. Bogy, L. Lin, *ACS Nano* **2019**, *13*, 7107.

[11]	G. M. Sessler, Ed., *Electrets*, Vol. 33, Springer, Berlin, Heidelberg **1987**.

[12]	T. Tanaka, G. C. Montanari, R. Mulhaupt, *IEEE Transactions on Dielectrics and Electrical Insulation* **2004**, *11*, 763.

[13]	Y. Gong, K. Zhang, I. M. Lei, Y. Wang, J. Zhong, *Advanced Materials* **2024**, *36*, 2405308.
